# Decoding the glycan shield: Immune recognition and response to the HIV-1 envelope trimer

**DOI:** 10.1016/j.jbc.2026.111350

**Published:** 2026-03-06

**Authors:** Fathima Zahra Nawaz, Trevor M. Adams, Mariye Erol Demirturk, Fikri Y. Avci

**Affiliations:** Department of Biochemistry, Emory Vaccine Center, Emory University School of Medicine, Atlanta, Georgia, USA

**Keywords:** adaptive immunity, bnAb, envelope glycoprotein, glycoimmunology, helper T cells, HIV, N-glycosylation, T-carb

## Abstract

The HIV-1 envelope glycoprotein (Env) is essential for viral entry and infection of host cells. Composed of a trimer of the gp120–gp41 heterodimeric glycoproteins, the Env trimer is the primary target for neutralizing antibodies. Extensive research over the past 40 years has focused on developing advanced immunogens, specifically recombinant, native-like Env trimers and structure-guided, germline-targeting constructs, to elicit protective antibody responses. The Env trimer is encased by up to 90 N-linked glycosylation sites, whose occupancy effectively shields the underlying protein from immune surveillance. While it is well established that glycosylation of HIV-1 gp120 affects antibody responses in infected individuals and that many broadly neutralizing antibodies depend on glycan-specific epitopes, the capacity of Env-derived glycopeptides to act as unconventional CD4^+^ T-cell epitopes and shape helper T-cell responses remains comparatively underexplored. This review examines the adaptive immune responses triggered by HIV Env, with an emphasis on how Env glycosylation simultaneously constrains B-cell recognition and contributes to antigen processing and T-cell–mediated immune responses, aiming to lay the groundwork for future vaccine development and to inform strategies that elicit robust and lasting protection against HIV-1 infection.

AIDS, caused by HIV-1 (Fig. 1), remains a leading cause of death from infectious diseases. Since the onset of the pandemic more than 4 decades ago, AIDS has claimed over 40 million lives (1). Despite substantial advances in antiretroviral therapy and structure-guided vaccine design, the development of a prophylactic HIV vaccine capable of eliciting durable, broadly protective immunity remains an unmet global health challenge (2, 3, 4). Recent vaccine strategies have focused primarily on overcoming barriers to B-cell activation, whereas comparatively less attention has been paid to how immunogen design influences CD4^+^ T cell help, a key determinant of effective and sustained antibody responses (5, 6, 7).

**Figure 1:**
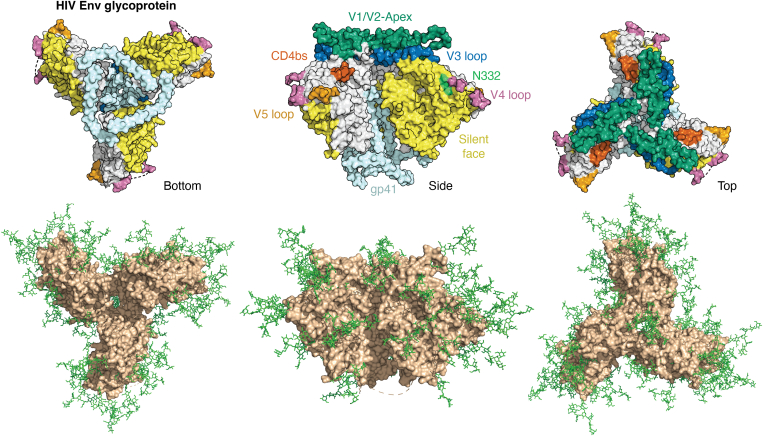
**Major antigenic regions of interest on the HIV Env glycoprotein.** In the *left panel*, the protein surface is colored to indicate antigenic sites. The gp120 subunits sit atop gp41 (*light blue*) at the base. In the *right panel*, the gp120 trimer protein backbone is depicted in *light brown*, and glycans are shown in *green*. Env, HIV envelope glycoprotein.

The extreme mutational diversity of HIV has been a significant obstacle in vaccine development, underscoring the need for an HIV-1 vaccine capable of generating an immune response resilient to this diversity (8, 9, 10). The primary target for vaccine development is the HIV envelope glycoprotein (Env) trimer. This heavily glycosylated surface protein is required for viral attachment to host cells *via* CD4 receptors and plays a key role in the viral replication cycle by mediating fusion of viral and cellular membranes (11). Many broadly neutralizing antibodies (bnAbs) that target distinct regions on the HIV-1 envelope recognize Env glycans (12, 13), highlighting glycans as both essential structural features of Env and central determinants of antibody-mediated neutralization (14, 15).

The Env protein is organized as a trimeric complex of gp120–gp41 heterodimers and is stabilized by weak noncovalent interactions (16). Synthesized initially as the gp160 polyprotein precursor, Env undergoes post-translational cleavage to yield the gp120 and gp41 subunits. The gp120 subunits are heavily glycosylated, with 23 to 26 N-linked glycosylation sites contributing to more than half of the protein's mass (17, 18). These N-linked glycans play critical roles in the viral life cycle, including assisting in protein folding, facilitating infectivity by binding to lectin receptors on immune cells, and aiding in immune evasion (19, 20, 21, 22, 23). Env glycans are widely regarded as a defining immunological barrier in vaccine design, exploited by pathogenic viruses to evade neutralizing antibody responses (24, 25). Accordingly, Env glycans influence both antibody accessibility and antigen processing.

Substantial efforts have been devoted to eliciting protective antibody production through the design of sophisticated immunogens, such as recombinant, native-like Env trimers (26). Reviews on contemporary immunogen design and germline targeting provide comprehensive summaries of how these strategies aim to engage rare neutralizing B cell precursors and overcome glycan-mediated barriers (25, 27, 28). Peptides are essential components of T-cell epitopes associated with major histocompatibility complex (MHC) binding and T-cell recognition. Research over the past decade has demonstrated that synthesized glycopeptides or naturally glycosylated immunogenic glycopeptides, derived from processed glycoproteins, can bind to MHC molecules (29, 30, 31, 32, 33, 34). Recent work reviews the mechanisms by which glycoprotein-derived epitopes are processed and recognized by the adaptive immune system, underscoring the importance of antigen structure and glycan context for T-cell engagement (14, 24). T-cell recognition of glycosylation-dependent epitopes occurs following natural exposure to glycoproteins, with both the glycan structure and the glycosylation site being critical for effective T-cell engagement (35). These findings indicate that glycosylation can directly contribute to adaptive immune recognition rather than serving solely as a passive shield. While it is well established that the glycosylation of HIV Env affects the antibody response in infected individuals and that many bnAbs target glycan-dependent epitopes, the contribution of Env glycans to shaping CD4^+^ T-cell responses has been comparatively underexplored (36, 37, 38).

CD4+ helper T cells are essential for driving antibody class switching, affinity maturation, and the effector function of antibodies against HIV (39, 40, 41). The generation of bnAbs particularly depends on affinity maturation and somatic mutations, which require CD4^+^ T cell help (41). Within antigen-presenting cells (APCs), Env glycan moieties survive antigen processing, leading to the presentation of glycopeptide epitopes by MHC II to CD4^+^ T cells (34). Thus, Env glycans may simultaneously impede germline B-cell receptor (BCR) engagement while contributing to helper T-cell epitope formation through MHC class II presentation (24, 42). Understanding the essential role of helper T cells in B-cell activation would lead to significant advances in the future development of glycan-based vaccines against HIV. Given the importance of structure-guided design in HIV vaccine development, this review focuses on the fundamental aspects of glycoimmunology and structural biology that underpin glycans in the HIV envelope trimer and their role in T-cell–mediated immune responses. It also explores potential approaches to harness glycan targeting for HIV vaccine design.

## The HIV-Env

### Structural organization of the HIV-1 Env trimer

**Figure 2:**
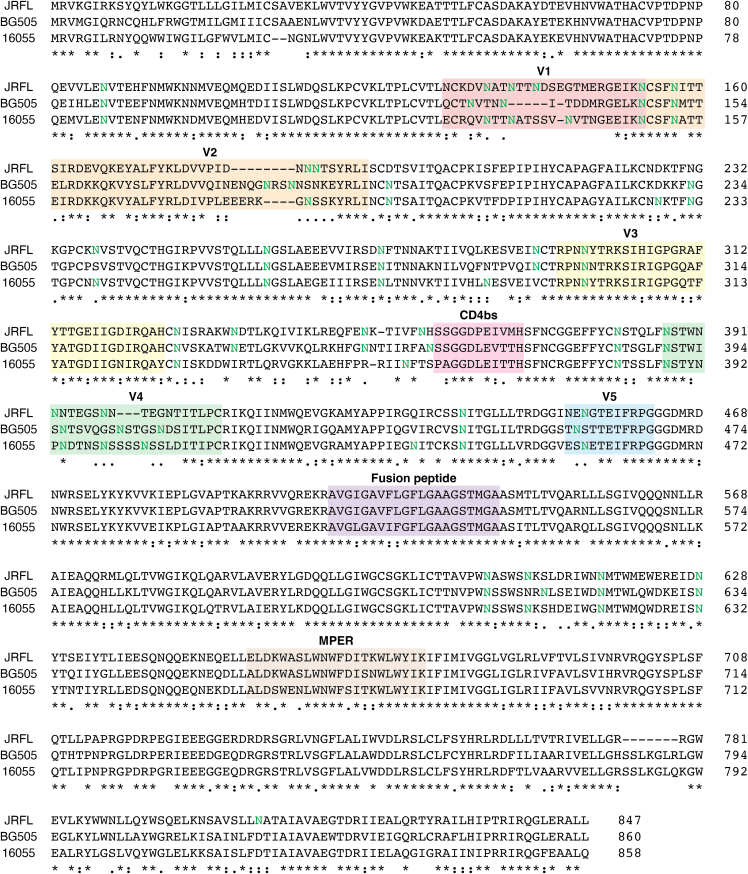
**Multiple sequence alignment of three HIV-Env protein sequences *via* Clustal Omega (1.2.4).** Selected sequences are from clade A (BG505), clade B (JRFL), and clade C (16055). Glycosylated asparagines are bolded in *green*. Antigenic regions of interest are marked and use the same color scheme as in Figure 1. Env, HIV envelope glycoprotein.

The HIV-Env trimer is a glycoprotein with a dense carbohydrate coat that serves as a “glycan shield,” protecting conserved peptide epitopes from immune surveillance through host-like N-glycans. Antigenic regions include the CD4-binding site (CD4bs), the V1–V2 apex, the V3 oligomannose patch, the outer domain oligomannose patch, the gp41–gp120 interface, and gp41 itself (Fig. 2) (43). BnAbs compete for similar sites in these regions, as demonstrated by bnAb cross-competition data (43).

Many modifications have been made to stabilize the trimer: a disulfide bond ("SOS") between residues 501 in the gp120 subunit and 605 in the gp41 subunit; an isoleucine-to-proline substitution at position 559 in the first heptad repeat of gp41 to prevent helix formation and lock the protein in the prefusion state; a modified furin cleavage site to ensure complete precursor cleavage between gp120 and gp41; and the deletion of the membrane-proximal external region (MPER) of gp41 to improve homogeneity and solubility (44, 45). The BG505 SOSIP.664 trimer closely resembles the native viral Env in both structure and antigenicity (44). The availability of the SOSIP trimer enabled the structural determination of numerous additional bnAb epitopes, including the ones requiring quaternary structures, such as those found at the trimer apex and the gp120–gp41 interface (46). More recently, native, flexibly linked trimers have emerged to produce native-like trimers without necessitating the use of furin (46). Cryo-EM and X-ray crystallography studies of the Env trimer have shown that the gp120 subunits adopt a globular conformation and assemble into a trimer through crucial interactions in the V1, V2, and V3 domains. The gp41 domain forms a pedestal, upon which the gp120 subunits are positioned, resulting in a mushroom-like structure for the functional trimer (36, 47, 48, 49).

### The HIV-Env glycan shield

**Figure 3:**
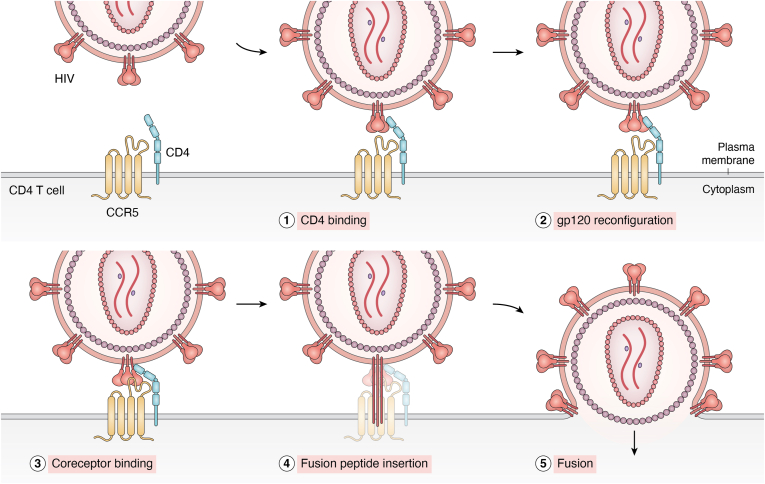
**HIV entry into a CD4 T cell.** This diagram illustrates the stepwise process of HIV entry into a CD4+ T cell. (1) HIV Env binds to the CD4 receptor on the host cell surface. (2) This binding induces a conformational change in Env (3), exposing a binding site for the coreceptor. Binding to the coreceptor triggers further conformational changes, leading to the insertion of the gp41 fusion peptide into the host membrane (4). This enables the viral and host cell membranes to fuse (5), allowing the viral core to enter the host's cytoplasm and start infection. Env, HIV envelope glycoprotein.

The dense N-glycosylation of HIV-Env is highly conserved and an important consideration in immunogen design (Fig. 3) (24), and overcoming these glycan barriers is seen as a key requirement for an effective immune response (50, 51). These dense sugar patches create a “canopy” effect where the flexibility of the glycans is reduced because of the preponderance of glycan–glycan interactions. Although this glycan canopy cannot be directly resolved by crystallography or cryo-EM, signal-to-noise filtering can be used to estimate its volume within a space-filling model (52). This glycan canopy can be viewed as a network of glycan–glycan interactions, with distinct regions of glycosylation forming interconnected topological microdomains (53).

Notably, the N-glycans of monomeric HIV-Env can be processed differently from those of the trimer. This distinction is exemplified by the trimer-associated mannose patch, which is processed into more complex N-glycans in the absence of trimerization (54, 55, 56). On the other hand, the intrinsic mannose patch forms regardless of the multimeric state (56). The trimer-dependent nature of the trimer-associated mannose patch likely results from monomer–monomer interactions that restrict access by glycan-processing enzymes, such as glycosyltransferases and glycoside hydrolases. The high density of glycans also plays a role in this N-glycan processing restriction, and structural rearrangements of proximal N-glycans can compensate for the loss of individual glycan sites (57). Thus, while numerous studies of monomeric HIV-Env N-glycan microheterogeneity are valuable, the data should be interpreted with caution (58, 59, 60, 61, 62, 63, 64).

### HIV-Env diversity and N-glycan metaheterogeneity

The site-specific N-glycosylation of engineered soluble HIV trimers has been extensively characterized using various mass spectrometry approaches. High-resolution studies (65, 66, 67, 68, 69, 70, 71, 72, 73) have primarily focused on clades A, B, and C, which together account for 40% to 70% of global HIV infections (note that estimates vary widely (74, 75)), with clade C contributing up to half of total infections. However, clades exhibit strong geographic clustering. For instance, clades A and B account for ∼60% to 85% of infections in Western Europe and North America, but less than 25% in sub-Saharan Africa, and around 6% in West and Central Africa. In Southern Africa, clade C makes up the large majority (>90%) of HIV infections. The transmission dynamics of these subtypes are influenced by key populations at heightened risk for HIV and their access to care, both of which vary across different regions. Many of the fine details of HIV subtype differences are not known; there is little evidence to suggest that the prevalence of HIV subtype C in India, Ethiopia, and Southern Africa is due to differences in viral fitness rather than sociopolitical phenomena impacting transmission dynamics (76).

Other subtypes, such as CRF01_AE (most prevalent in East/Southeast Asia) and CRF02_AG (dominant in West and Central Africa), remain less characterized, with available data mostly limited to site occupancy (18, 77) *via* enzymatic glycosylation site labeling schemes (78). While site occupancy data do not provide information on the specific glycoforms that are found on sites of N-glycosylation, when combined with alignment data, they are instrumental in determining which sites of glycosylation are essential to maintain for viral fitness. A 2018 study by Yu *et al.* (79) is an example of this, where 94 gp120 monomers were assessed for glycan site occupancy and antigenicity. The near-ubiquitous conservation of glycan occupancy at specific sites (N88, N262, and N448) suggests that some glycosylation sites are critical in maintaining viral fitness, possibly because of protein stability, epitope masking, or another important function. In addition, glycan redundancy was observed for some pairs of neighboring sites, such as N156/N160 and N295/N301, where subtypes are frequently missing one member of a pair but never both. In these cases, it may be necessary to knock out both sites to expose the potential antigenic site beneath the glycan coat. Site occupancy studies have also helped compare manufacturing methods for Env antigen production (77) and will remain a valuable tool for characterizing new subtypes with higher throughput.

While glycans are critical for immune evasion, less is known about how glycan shield features early in infection impact the downstream development of neutralization breadth in people living with HIV. It does not appear that the size of naturally occurring general glycan holes in Env from early infection is correlated with neutralization breadth (80). This contrasts with the promising approach of using engineered immunogens with targeted glycan holes in recent germline-targeting vaccine efforts (see *Germline targeting* section). For example, the extent of surface exposure from targeted N-glycan sequon knockouts correlates with autologous neutralizing antibody responses (81). Unique glycan profiles have also been associated with discrete modes of transmission, which is a noteworthy consideration for global transmission dynamics (82).

## BnAbs and their recognition of HIV

BnAbs are a specialized class of antibodies that neutralize a wide range of HIV strains by targeting conserved, mutation-resistant epitopes on the HIV Env (83). These epitopes are frequently shielded by a dense array of N-linked glycans, which bnAbs engage through precise structural adaptations, either directly or by navigating around them (2, 84). Functionally, bnAbs block viral entry by neutralizing free virions. In addition, some bnAbs mediate Fc-dependent effector functions, including antibody-dependent cellular cytotoxicity, contributing to the clearance of infected cells. Notable bnAbs, such as b12, 3BNC117, PGT121, and 10–1074, have demonstrated antibody-dependent cellular cytotoxicity activity *in vitro* and *in vivo*, underscoring their dual roles in viral neutralization and immune-mediated clearance (85, 86, 87).

The natural development of bnAbs during infection is lengthy, typically requiring several years and repeated exposure to evolving viral antigens. This process is characterized by extensive somatic hypermutation, often resulting in antibodies that are more than 30% diverged from their germline immunoglobulin sequences (88). Such high levels of affinity maturation highlight the complexity of generating bnAbs through immunization and emphasize their relevance in therapeutic and vaccine development.

BnAbs target multiple conserved regions of the HIV Env trimer, including the CD4bs (*e.g.*, VRC01), the V1/V2 glycan apex (*e.g.*, PG9, PG16), the V3 glycan patch (*e.g.*, PGT121), the gp120–gp41 interface (*e.g.*, PGT151), and the MPER of gp41 (*e.g.*, 10E8) (36, 89, 90, 91, 92, 93). Many of these antibodies are glycan dependent, engaging conserved glycosylated motifs, such as the N332 or N334 supersite, the V1V2 apex, and the silent face of Env (Table 1) (17, 94).

**Table 1:** Summary of HIV-1 bnAbs: epitope targeting, glycan specificity, and structural mechanisms of recognition

bnAb	Targeted epitope	Glycan specificity	Mechanism of recognition	References
PGT121 family (PGT121–123, 10–1074, BG18)	V3 glycan patch (N332, N301, ±N137/N156)	High-mannose and complex-type glycans	Engages glycans and peptide elements, including the GDIR motif	(99, 176)
PGT124 family	V3 loop base N332 glycan	Man_8_GlcNAc_2_ glycan (Man_8_)	Engages glycan linked to N332 and a small protein segment at the V3 loop base	(176)
PGT128 family (PGT125–131)	V3 glycan patch (N332, N301)	High-mannose (Man8, Man9)	Long CDRH3 loop penetrates glycan shield to access peptide–/glycan interface	(99, 177, 178)
PGT130/PGT131	V3 glycan patch (N334)	High-mannose	Similar to PGT128, different glycan preference	(177)
PGT135 family (PGT135–137)	Base of V4 loop (N295, N386, N392)	High-mannose	Binds N332 and adjacent glycans	(176, 177)
2G12	Silent face of gp120 (N295, N332, N339, N392)	High-mannose	Unique domain-swapped Fab binds terminal mannose without peptide contact	(112)
PG9/PG16	V1/V2 apex (N156, N160)	High-mannose	Long CDRH3 loop penetrates apex glycan shield to reach protein	(100)
PGT145	Trimer apex (N160)	High-mannose	Recognizes quaternary trimeric conformation with specific glycan presentation	(94)
PGT151	gp120–gp41 interface (N611, N637)		Recognizes cleaved Env, binds both peptide and glycan at the interface	(178)
438-B11	N332/V3 glycan supersite (N301, N332)	High-mannose	Unique angle of approach; binds GDIR motif and flanking glycans	(179)

Glycan-reactive bnAbs either bind isolated glycan motifs or composite glycopeptide structures, turning HIV's glycan shield—a major mechanism of immune evasion—into a targetable vulnerability. For instance, the bnAb 2G12 binds to high-mannose glycans on multiple Env sites and exhibits crossreactivity with the influenza hemagglutinin glycoprotein, despite significant differences in protein sequence (95). The successful elicitation of such antibodies requires activation of rare naive B cell precursors and their recruitment into germinal centers (GCs), where they must outcompete B cells targeting more immunodominant but non-neutralizing epitopes (96).

The dense glycan shield of the HIV Env glycoprotein was initially considered solely a mechanism for evading host immune responses. However, it is now recognized to be a critical target for vaccine design (97, 98). Several bnAbs, including PG9, PGT128, and members of the PGT121–123/PGT133–134/10–1074, PGT125–128/PGT130–131, and PGT135–137 families, recognize and exploit glycan-dependent epitopes on the HIV Env glycoprotein (37, 48, 90, 99). Structural analyses, such as crystal structures of PGT128 and PGT135 bound to gp120 domains and PGT122 in complex with the BG505 SOSIP.664 trimer, have revealed how multivalent recognition of glycans and underlying protein surfaces enhances breadth and potency (37, 48, 100). Despite heterogeneity in glycan processing, conserved sites, such as N332, remain accessible because of their conserved motif of terminal α-linked mannose residues.

To elicit glycan-reactive bnAbs through vaccination, immunogens must effectively engage specific germline precursors, which are often characterized by long heavy chain complementarity-determining region 3 loops capable of penetrating the glycan shield (96, 101). For instance, initiating PGT121-class responses requires a closed N332 glycan-binding face and an open surface to accommodate or avoid neighboring glycans. Strategic glycan removal, such as deleting the N137 glycan, may improve early germline targeting, though its impact on later maturation stages remains unclear (102, 103).

Sequential immunization strategies, which present a series of Env immunogens mimicking the virus's natural evolution, can guide a rare somatic hypermutation and clonal selection, ultimately guiding the maturation of high-affinity bnAbs (96, 104, 105). Native-like Env trimers preserving glycan architecture are essential to promote the correct angle of approach and durable bnAb responses. These findings underscore the immune system’s adaptability in targeting HIV's heavily glycosylated Env and provide a blueprint for structure-based vaccine design (106, 107).

Despite these advances, significant challenges remain. HIV's extraordinary sequence diversity, driven by high mutation and recombination rates, poses a major hurdle for vaccine development (83). Potent bnAbs achieve their exceptional breadth and potency through atypical features, including unusually long heavy chain complementarity-determining region 3 loops, extensive somatic hypermutation, and the acquisition of rare but functionally critical “improbable mutations” (101). These mutations are often disfavored during B-cell development because of central and peripheral tolerance checkpoints, leading to deletion or anergy of bnAb-precursor B cells, particularly those targeting glycan-dense epitopes like the V2 apex, V3 glycan patch, and MPER (93). In addition, the dense glycosylation of the HIV Env trimer and the immunodominance of non-neutralizing epitopes further hinder the effective induction of bnAbs by natural infection or conventional vaccination strategies (2).

A key rate-limiting step in bnAb elicitation is the sustained engagement of rare precursor B cells within GCs, allowing for the accumulation of the somatic mutations required for high-affinity, broad neutralization. In natural HIV infection, bnAb lineages often display polyreactive or autoreactive features typically eliminated during the B-cell maturation process.

These approaches must preserve GC re-entry and clonal persistence to enable the breadth and potency characteristic of bnAbs—achieving in months what typically takes years during chronic infection. An essential goal in vaccine design is therefore to develop immunogens capable of engaging BCRs from multiple bnAb precursor lineages to initiate broader polyclonal responses to conserved Env epitopes than typically occur during natural infection, through processes such as sequential immunization strategies (108).

## B-cell activation and differentiation in HIV infection

During HIV infection, the viral Env trimer, a heavily glycosylated and structurally flexible antigen, not only serves as the primary trigger for B-cell activation but also poses significant challenges for effective antibody recognition (49). Dense glycan shielding, conformational masking, and the limited number of Env spikes on the virus surface hinder BCR engagement and crosslinking, delaying the development of strong neutralizing antibody responses. As a result, the production of effective antibodies often requires prolonged GC reactions and extensive somatic hypermutation, with some antibody lineages evolving the ability to recognize unique glycan–protein composite epitopes characteristic of HIV Env (3, 49).

Structural analyses have elucidated the mechanisms by which bnAbs target glycan–protein composite sites on the closed, prefusion form of the HIV Env trimer (3). Insights into bnAb development, tracing their progression from naive B cells through prolonged somatic hypermutation, have shaped vaccine strategies focused on both antibody lineage and specific epitopes. These strategies seek to activate rare bnAb precursor B cells and steer their evolution using a series of designed immunogens.

### B-cell development and maturation

B cells originate in the bone marrow, where they undergo V(D)J recombination, a process that assembles variable (V), diversity (D), and joining (J) gene segments, to produce a diverse repertoire of BCRs. This diversity, estimated to recognize more than 5 × 10^13^ distinct antigens, is essential for broad immune surveillance. Following successful recombination, immature B cells express surface IgM and exit the bone marrow as transitional B cells. These transitional B cells migrate to peripheral lymphoid tissues, such as the spleen and lymph nodes, where they continue to mature into naive B cells (109).

### Activation and GC reaction

Upon encountering an antigen in these secondary lymphoid organs, naive B cells can be activated in a T-cell–dependent or T-cell–independent manner, depending on the nature of the antigen and the context of the immune response (110). In T-cell–dependent responses, activated B cells localize to GCs, specialized microenvironments within B cell follicles that facilitate the generation of high-affinity, antigen-specific responses.

Within GCs, B cells undergo iterative cycles between (111)

• Dark zone—proliferation and somatic hypermutation of BCR genes.
• Light zone—selection by T follicular helper (Tfh) cells, favoring B cells with the highest affinity for antigen.
• Successful affinity maturation produces either long-lived plasma cells, which secrete antibodies, or memory B cells, which provide rapid recall responses upon reexposure (112).

### Impact of HIV on B-cell responses

In chronic HIV infection, sustained antigen exposure and systemic immune activation disrupt GC homeostasis (113). This dysregulation can

• Impair coordination between B cells and Tfh cells.
• Skew differentiation away from effective memory B cell and plasma cell formation.
• Promote B-cell exhaustion and functional decline.
• The mechanisms underlying these changes remain incompletely understood but likely involve persistent HIV antigen presentation, altered cytokine environments, and structural damage to lymphoid tissues (113). These disturbances limit the generation of bnAbs in most individuals, with only a small subset developing potent bnAb responses after years of infection (83).

## Germline targeting and glycan engineering

A rapidly evolving area of study is the germline targeting of specific subpopulations of naive B cells to recapitulate observed broadly neutralizing responses (also recently reviewed elsewhere (25, 114). Population-level analyses of antibody development against the CD4bs have elucidated binding modes that are recapitulated by distinct antibody–interface interaction features, which are associated with subsets of B cell precursors (115) or with the use of specific V_H_ genes (116). Many studies have sought to consistently recapitulate these and other modes of binding across a variety of Env antigenic sites by using germline-targeting approaches (108, 117, 118, 119). These efforts explicitly acknowledge that native, fully glycosylated Env is poorly immunogenic for bnAb precursor B cells and must be artificially modified to overcome natural barriers to antibody lineage initiation (3).

One key strategy in germline targeting is the use of engineered glycan holes to generate immunogens that bind to these germline B cells. Their activation and subsequent activation generate progeny B cells, which will mutate their receptors and increase the odds of a bnAb-forming lineage. Antibody–antigen binding profiles are altered when glycan holes are introduced (120). The development of VRC01, a potent vaccine-induced bnAb, is being investigated using various germline-targeting strategies (114). Many of these approaches involve knocking out N-glycosylation sites, particularly N127 in clade C strain 426c. This modification can expand the range of VRC01-class antibodies to which the engineered Env can bind (121). Removal of specific glycans is a requirement for germline reactivity in certain constructs (such as N276 in the eOD-GT8 construct), although this glycan’s broad conservation likely means that a glycosylated booster would be needed to develop reactivity toward nonengineered Env (105). Env-antibody coevolution studies helped develop a strategy to stimulate the RHA10 lineage in which a glycan hole is transiently introduced in prime immunogens and then reintroduced in boosts (122). Importantly, these strategies are optimized to initiate bnAb lineages *via* BCR engagement and do not directly account for the parallel requirement for sustained CD4^+^ T-cell help during prolonged GC reactions (5, 7, 123).

Expression of Env in MGAT1-knockout cells produces Env trimers with a homogenous high-mannose glycan profile, mainly containing five mannoses (though more mannoses are present when glycan processing is incomplete), which enhances binding to select precursors (124). Glycan occupancy engineering, introducing N-glycan sites and improving occupancy in the V1-loop of the V3-glycan–targeting N332-GT5 construct, reduces binding by undesirable epitope-specific Fabs (125). Some finer details of the glycan shield's effects, such as charge, size, and terminal sugars, can be assessed by using glycosidases to modify existing glycan moieties (126). Notably, these glycan engineering approaches have largely been evaluated for surface recognition by BCRs, with comparatively little consideration of how altered glycosylation influences antigen processing, MHC class II presentation, or the availability of cognate CD4^+^ T-cell epitopes (24, 42). As a consequence, glycan deletions that enhance germline B-cell activation may simultaneously remove glycan-containing determinants that could otherwise contribute to helper T-cell responses, revealing a potential trade-off that has not been systematically explored.

Altogether, glycan occupancy, heterogeneity, and prime-boost strategies are critical considerations in vaccine design. Extending these design principles to account for both BCR engagement and CD4^+^ T cell epitope generation may be necessary to fully support bnAb maturation.

## T-cell–mediated regulation of B-cell responses in GCs

Taken together, these considerations suggest that even optimally engineered Env immunogens capable of activating bnAb-competent B cells will fail to elicit durable broadly neutralizing responses unless they also support effective cognate CD4^+^ T-cell help within GCs, prompting closer examination of how T-cell–mediated regulation shapes B-cell fate decisions during HIV vaccination.

CD4^+^ T-cell help is critical for the initiation and maturation of B-cell responses within GCs, where high-affinity antibodies are produced. In the early stages of a humoral response, Tfh cells facilitate the recruitment of antigen-specific B cells into GCs and support their subsequent affinity maturation through cycles of somatic hypermutation and selection (5, 127). This interaction is essential for generating effective antibody responses against diverse pathogens.

One key challenge in vaccine development, particularly in the context of HIV, is B-cell immunodominance; a phenomenon where immune responses are skewed toward dominant, often suboptimal epitopes. This is especially prominent in responses to the Env, where Tfh cells play a central role in GC dynamics, yet their impact on modulating immunodominance remains unclear (128, 129, 130). While enhancing Tfh cell help may theoretically promote the expansion of rare B cell clones capable of producing bnAbs, it could also intensify existing immunodominance by preferentially supporting more abundant, lower-affinity B cells. This presents a significant obstacle in the design of HIV vaccines aimed at bnAb induction.

Supporting this, studies in nonhuman primates have demonstrated that increased frequencies of GC B cells and neutralizing antibodies correlate with the presence of antigen-specific Tfh cells following Env immunization (129, 131, 132). Similarly, in HIV-infected individuals, the emergence of bnAbs is associated with elevated levels of circulating Tfh cells (133, 134). However, effective Tfh-mediated help requires that antigen-specific B cells can present cognate T-cell epitopes *via* MHC class II molecules; a process potentially hampered by the extensive glycosylation of Env. Indeed, in both C57BL/6 mice and rhesus macaques, MHC class II responses to Env were restricted to a limited number of unglycosylated regions (135, 136), suggesting that much of the Env sequence is poorly suited for efficient T-cell engagement.

Further illustrating the importance of T-cell help, antibody responses to core gp120 constructs in small-animal models were weak but could be substantially enhanced by incorporating the universal pan-DR epitope, a broadly recognized T-helper epitope, into the immunogen design (137, 138). This underscores the combined limitations of restricted CD4 T-cell epitope availability and the low precursor frequency of bnAb-competent B cells, both of which can constrain access to adequate Tfh support (128, 139). Together, these findings underscore the importance of elucidating the interplay between T-cell help and B cell selection in immunodominant settings—a crucial step toward identifying predictive markers of successful, protective humoral immunity.

## Recognition of HIV-1 Env by APCs for processing and presentation

The first step in antigen processing begins with the uptake of HIV-Env virions by dendritic cells (DCs) and macrophages (140). These are thought to be the first cells that interact with the virion during viral transmission. Thus, their relationship and interactions with transmission/founder HIV virions are critical to early viral response. DCs can facilitate the transmission of HIV to uninfected CD4^+^ T cells (141). The first step in antigen processing begins with the uptake of HIV-Env virions by DCs and macrophages (140). These are thought to be the first cells that interact with the virion during viral transmission. Thus, their relationship and interactions with transmission/founder HIV virions are critical to early viral response. DCs can facilitate the transmission of HIV to uninfected CD4^+^ T cells (141). Therefore, elucidating the interactions between virions and DCs is essential not only for understanding HIV-Env antigen presentation but also for viral infection and progression. Much of our knowledge of HIV–DC interactions is studied through the lens of viral progression.

While the hallmark receptors for HIV-Env binding are CD4 and CCR5, other receptors can bind independently. C-type lectin receptors (CLRs) are of particular importance as binding partners, and glycan microarray data show overlapping binding preferences toward different carbohydrate subgroups (142). The DC-specific ICAM-3 grabbing nonintegrin (DC-SIGN), a group II CLR, and a type II transmembrane protein (143), is of great importance as its high expression in immature DCs in mucosal tissues promotes infection through viral attachment to neighboring CD4^+^ T cells after migration to secondary lymphoid organs (142, 144). This interaction is facilitated by DC-SIGN binding to ICAM-3, which is highly expressed on resting T cells (145). Glycan microarray data suggest that the carbohydrate recognition domain of DC-SIGN has broad, dual specificity (146). DC-SIGN can bind a variety of high-mannose glycans as well as compact fucosylated O-glycans, such as Lewis^x^ and Lewis^a^ O-glycans, demonstrating DC-SIGN’s ability to broadly recognize molecular patterns from yeast, protozoa, viruses, and other pathogens. A common mannose substructure found within high-mannose N-glycans, Manα1–3(Manα1–6) Manα, binds to DC-SIGN. This interaction is facilitated by Ca^2+^ and a “blocking” phenylalanine residue, which prevents DC-SIGN from recognizing core GlcNAc residues. However, GlcNAc (and presumably other moieties) can be accommodated when attached externally to this trimannose structure (147), enabling DC-SIGN’s broad specificity.

While DC-SIGN does not efficiently facilitate the infection of DCs by HIV, it may be involved in promoting the lysosomal degradation of viral products when expressed in both B cells and DCs (147). DC-SIGN is not necessary for the uptake of virions by DCs (148) but is sufficient to cause uptake in fibroblasts when expressed exogenously (146). Interestingly, DC-SIGN is upregulated in HIV-infected DCs through a mechanism involving a negative factor, demonstrating the importance of this pathway in facilitating viral transmission (148).

Other DC-associated lectins have been shown to bind HIV-Env, including mannose receptor (MR) and langerin. Langerin, which is expressed exclusively by Langerhans cells, binds to the HIV-Env and, unlike DC-SIGN, facilitates viral internalization and degradation *via* Birbeck granules, thereby preventing viral transmission (149). MR, expressed by DCs and macrophages, also has a broad specificity toward a variety of glycans but appears to have a particular affinity for α1,2-linked mannose residues, which are found at the terminus of high-mannose structures with >5 mannoses (150). In cultured monocyte-derived macrophages, most HIV–macrophage interactions are mediated by the MR (151). Although the initial interaction between HIV and MR appears to facilitate infection of macrophages and T cells, MR functions as a restriction factor in the absence of HIV viral protein R and a negative factor by binding to and degrading pathogens coated with high-mannose residues (152). Langerin recognizes as little as one mannose residue, with evidence suggesting that nonreducing-end α1,3-linked mannose residues may further enhance its binding affinity (153).

Other DC CLRs, such as DEC-205 and LSECtin, are not known to interact directly with HIV-Env. LSECtin can bind to envelope proteins from other viruses, such as filovirus and Ebola; however, it does not recognize high-mannose glycans, and direct binding assays have shown little to no interaction with HIV-Env. DEC-205 (also known as CD205) identifies apoptotic cells, facilitating antigen uptake and presentation *via* both the MHC I and MHC II pathways (154). However, its binding partners remain elusive, and although it is a CLR-family protein, it lacks the common binding motifs responsible for mannose binding in related proteins (155). While beyond the scope of this review, nonhuman lectins should also be recognized as useful tools for detecting and inhibiting HIV, with coupling to antibodies offering a novel basis for therapeutics (156, 157).

## Antigen processing and presentation pathways of HIV-Env

Understanding the relationship between HIV and its recognition by the adaptive immune system is key to understanding what epitopes are actually presented for recognition (158). Resting, immature DCs in mucosal tissues encounter the virus early in HIV infection, thus playing a critical role in the early stages of the disease. Generally, antigen processing for MHC I complexes occurs *via* the proteasome on newly synthesized viral proteins in infected cells. Some of the generated epitopes are presented *via* transporter associated with antigen presentation–independent pathways and others *via* transporter associated with antigen presentation–dependent pathways in an epitope-specific manner (159). These peptides are generated through cleavage by metallopeptidases and proteasomes (160). Nonglycosylated HIV-Env peptides containing N-glycosylation sequons are identified as class 1-restricted epitopes but not with a glycan attached (161).

**Figure 4:**
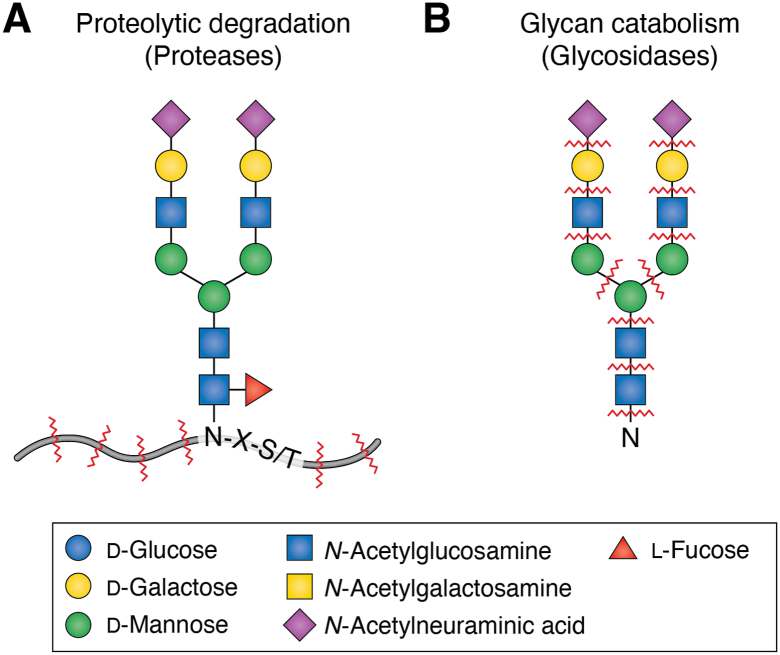
**Major features of N-glycopeptide catabolism in endolysosomal compartments.** N-glycoprotein degradation can create a wide range of potential glycopeptide products because of the varying processivity of degradation of peptide bonds, peptide–glycan bonds, and glycan–glycan bonds.

In contrast, antigen processing for MHC II complexes occurs when internalized exogenous virus is taken up by cells that are not necessarily infected. DC-SIGN–mediated internalization of viruses by DCs is thought to facilitate infection by prolonging viral half-life through trafficking to a low-pH, nonlysosomal compartment (162). The exact mechanism of this internalization is unclear. Primary infection occurs *via* classical receptor–mediated fusion, activated by virion binding to CD4 and CCR5–CXCR4; however, the exact site of fusion (at the plasma membrane or *via* an endocytic pathway) remains incompletely understood. Macropinocytosis has been proposed as a mechanism of uptake in primary human macrophages (163) and in brain microvascular endothelial cells (164). Once encapsulated within an endosomal compartment, a variety of mechanisms process proteins into antigens for presentation, which, for glycoproteins, involves digestion of both the protein and the glycan moiety (Fig. 4). Reductases such as γ-interferon-inducible lysosomal thiol reductase reduce disulfide bonds to facilitate protein unfolding. Cathepsins—a family of proteases—broadly and nonspecifically degrade polypeptides, generating potential binding partners for MHC class II molecules. Some evidence suggests that cathepsin cleavage sites can be incorporated into the HIV-Env to prevent the generation of amenable epitopes during antigen processing (165). The breakdown of N-glycans is not necessary for proteolysis, as partially intact, proteolyzed glycans can be found in mannosidosis, a lysosomal storage disease caused by a deficiency of mannosidase activity (166).

In addition, lysosomal nonreducing end glycosidases can act in the presence of asparagine attached at the reducing end (167). However, proteolysis is necessary for cleavage of the N-glycosidic bond, as lysosomal aspartylglucosaminidase requires free α-amino and α-carboxyl groups for activity (168), keeping aspartylglucosaminidase from contributing significantly to glycoprotein antigen processing. Bidirectional N-glycan degradation is thought to proceed after cleavage of the peptide-glycan bond. Recent studies on mucin glycodomain catabolism indicate that this also occurs with O-glycans (169). Further research at the intersection of endolysosomal glycopeptide processing and MHC II presentation holds excellent potential for developing novel therapies and vaccines.

## T-cell responses to HIV-Env: Antigen presentation and effector functions

**Figure 5:**
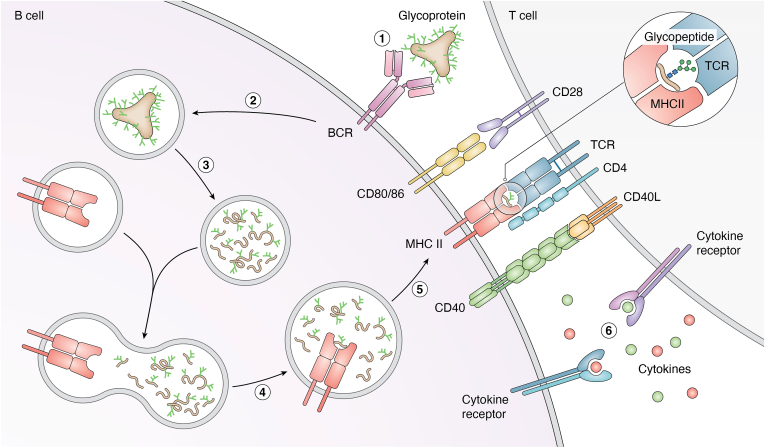
**Molecular interactions between a B cell and a CD4^+^ T cell during HIV Env presentation and T-cell activation.** (1 and 2) Antigen binding and internalization by the BCR. Depicted here are high-mannose structures crosslinking two Fabs on BCR and internalized by receptor-mediated endocytosis into the B cell. (3) Antigen processing. The internalized antigen is processed in the endolysosomes into smaller peptide and glycopeptide fragments. (4) Class II MHC loading. Processed MHC II binding peptides and glycopeptide epitopes are loaded onto MHC class II molecules (shown here with a glycopeptide). (5) Antigen presentation to CD4+ T cell. The (glyco)peptide–MHC II complex is transported to the B-cell surface, where it is recognized by the T-cell receptor (TCR) on a helper T cell. CD4 stabilized this interaction. (6) The immune synapse is established when CD40 on B cells engages with CD40 ligand (CD40L) on T cells, driving B-cell activation, proliferation, and isotype switching. Costimulatory signaling occurs through CD80/86 on B cells, binding to CD28 on T cells, ensuring full T-cell activation. Activated T cells secrete cytokines, including interleukin-2 (IL-2) and IL-4, which act on both T and B cells to promote clonal expansion and differentiation. In addition, cytokines critical for T follicular helper (Tfh) cell function—such as IL-6 and IL-21—enhance Tfh differentiation, germinal center formation, and B-cell affinity maturation. Together, these interactions yield a professional humoral immune response, characterized by class-switched, high-affinity antibody production and the generation of long-lived memory B and T cells. BCR, B-cell receptor; Env, HIV envelope glycoprotein; MHC, major histocompatibility complex.

Glycosylation can modulate T-cell recognition by either masking conventional epitopes through conformational changes or steric hindrance or by generating novel glycoepitopes that are specifically recognized by T cells (Fig. 5) (11, 34, 170, 171). Although glycosylation-dependent T-cell responses have been described in contexts, such as tumors, tuberculosis, and viral infections, they remain relatively underexplored in HIV, with only a few studies reporting T-cell recognition of HIV-derived glycoepitopes (34, 170). Recent studies of antigen processing and unconventional MHC ligands further emphasize that glycan-modified peptides represent a broader, emerging class of T-cell antigens than previously appreciated (172, 173).

In a recent study from our group, we identified and characterized a distinct CD4^+^ T-cell repertoire that recognizes explicitly a glycopeptide epitope from HIV-1 gp120 presented *via* the MHC class II pathway (34). This finding highlights a nonconventional MHC ligand and demonstrates that T cells can engage with glycosylated antigens beyond standard peptide epitopes. We refer to these glycopeptide-specific CD4^+^ T cells as T-carbs. They recognize not only the peptide backbone of gp120 but also its glycan modifications when displayed by MHC II, and this recognition is strictly glycan and MHC II dependent. Importantly, the nature and position of the attached glycans determine T-cell responsiveness, underscoring the role of carbohydrate moieties in shaping antigen specificity. This mode of recognition is consistent with emerging paradigms in glycoimmunology that position glycans as integral components of adaptive immune epitopes rather than passive modifications (42, 174).

T-carbs are highly immunogenic, capable of eliciting robust glycan-dependent cellular and humoral responses (31, 33, 34). Functionally, they enhance Env trimer–specific antibody development, including neutralizing antibodies, facilitate antigen uptake by APCs through the production of antibodies targeting glycan-dependent Env epitopes, and provide broad cross-clade help, as GpepIP-specific T cells recognize glycopeptide/glycan epitopes generated from heterologous Env variants despite high sequence variability. Phenotypically, glycopeptide epitope-specific T-carbs preferentially differentiate into Th2 (interleukin [IL]-4, IL-5, and IL-13) and Th17 (IL-17A, IL-17F, and IL-22) subsets, alongside elements of Th1 programming (interferon gamma, IL-2). These profiles are well suited to promoting B-cell help, antibody class switching, affinity maturation, and mucosal immunity—all critical for HIV vaccine efficacy. In contrast, the corresponding nonglycosylated peptide epitope induces a Th1-skewed response and is less potent in driving antibody breadth and function (34).

Our study further revealed that gp120 glycans survive APC antigen processing, yielding stable MHC II-bound glycopeptides. This finding challenges the traditional view of Env glycosylation solely as an immune shield. Instead, it positions Env glycans as conditionally immunogenic determinants that can contribute to productive helper T-cell responses. The antigenicity of the glycopeptide epitope is not attributable to autoreactivity, as its glycan structures differ substantially from host N-glycans, aligning with observations that glycan-targeting bnAbs rarely display autoreactivity, yet remain protective.

As a proof of principle, glycopeptide epitope priming, followed by Env trimer (BG505) boosting, induced tier 1 neutralizing antibodies and antibodies with enhanced functional capacity to mediate antigen uptake, effects not achieved with peptide priming or adjuvant alone. These results indicate that glycopeptide epitopes elicit more effective, functionally superior antibody responses than conventional peptide epitopes, supporting their potential utility as T-cell epitopes in rational immunogen design.

Collectively, these findings expand the known spectrum of pathogen-derived antigens that elicit T-cell–dependent B-cell responses to include glycopeptides. Incorporating such epitopes into immunogens could reliably elicit MHC II-restricted glycopeptide-specific T-cell help, thereby improving antibody quality, breadth, and durability. This approach addresses a key gap in current HIV vaccine design, which often prioritizes antibody induction without fully optimizing the T-cell help required for high-quality humoral immunity (25, 128).

Despite these advances, relatively little is known about how large N-linked glycans—such as those decorating HIV Env—affect antigen processing, MHC presentation, and T-cell receptor recognition. The emerging evidence that glycan-modified Env fragments can be effectively processed and presented challenges the traditional view of glycosylation as merely an immune shield (172, 173). Addressing this knowledge gap is particularly important because effective HIV prevention is generally thought to require both humoral and cellular immunity.

While pursuing both arms of immunity increases the complexity of vaccine design and complicates the identification of immune correlates of protection, strategic incorporation of glycopeptide epitopes offers a tractable means to couple Env-directed antibody responses with optimized CD4^+^ T-cell help, thereby synergistically enhancing Env-targeted vaccine efficacy through MHC II-restricted glycopeptide antigen presentation.

## Conclusions and future perspectives

Despite decades of research, HIV vaccine development remains challenged by the virus’s extraordinary genetic diversity, sophisticated glycan shield, and complex immune evasion mechanisms. While recent structure-guided and germline-targeting strategies have yielded important conceptual and experimental advances, achieving durable, broadly protective immunity remains an unmet goal (2, 3, 4). Recent advances in glycoimmunology and structural biology highlight the critical role of HIV Env glycans; not only as barriers to immunity but also as dynamic determinants of immune recognition, capable of shaping bnAb development and influencing antigen processing and presentation (13, 25). Further studies are needed to more fully define the extent and diversity of HIV Env glycoproteoforms and to capture these subtleties in greater detail.

A promising avenue lies in the targeted induction and optimization of glycopeptide-specific CD4^+^ T-cell responses (“T carbs”), which recognize glycosylated Env fragments presented *via* MHC class II pathways. Positioned alongside established B-cell engagement strategies, glycopeptide-specific helper T cells represent an underexplored but potentially enabling layer of vaccine-induced immunity. These glycopeptide-specific helper T cells differentiate into phenotypes that are particularly effective at supporting B-cell activation, antibody class switching, and mucosal immunity; key features for robust, protective humoral responses (5, 34). Incorporating glycopeptide epitopes may therefore complement germline-targeting immunogens by improving the quality, durability, and breadth of T-cell help available to bnAb-competent B cells.

Furthermore, structure-guided immunogen design, including native-like Env trimers that preserve glycan architecture and sequential immunization strategies, has demonstrated potential to guide rare bnAb precursor B cells through affinity maturation pathways despite stringent central and peripheral tolerance checkpoints (96, 114, 118). These approaches primarily address barriers at the level of BCR engagement, yet balancing immunodominance and ensuring sustained GC reactions with adequate Tfh cell support remain critical challenges (128, 131, 175).

Future HIV vaccine strategies can focus on a refined, multipronged approach that

• Integrates glycan engineering for germline B cell engagement with targeted induction of glycopeptide-specific CD4^+^ T-cell help, thereby supporting bnAb-competent lineages throughout GC evolution.
• Incorporates structure-based design principles to preserve native Env glycosylation patterns, enabling accurate mimicry of viral epitopes critical for bnAb elicitation.
• Utilizes sequential immunization protocols to drive B cell clonal evolution, enhancing the breadth and potency of antibody responses within a shortened time frame.
• Investigates the mechanistic foundations of glycan processing, antigen presentation, and T-cell receptor recognition of large N-linked glycans to refine immunogen design and immune engagement.
• Addresses immune dysregulation and B-cell exhaustion in chronic HIV infection by enhancing early T-cell help and GC dynamics.

By integrating insights from glycoimmunology, B-cell biology, and structural vaccinology, this targeted and mechanistically informed approach holds promise to overcome longstanding hurdles in HIV vaccine development. Focusing on tractable and specific immune mechanisms, particularly the rational engagement of glycopeptide-specific CD4^+^ T-cell responses, offers a complementary strategy to existing bnAb-focused paradigms, with the potential to streamline vaccine design, accelerate protective immunity, and ultimately yield a safe and effective HIV vaccine capable of controlling the global pandemic.

## Conflict of interest

The authors declare that they have no conflicts of interest with the contents of this article.
